# Application of three different coaching strategies through a virtual coach for people with emotional eating: a vignette study

**DOI:** 10.1186/s40337-020-00367-4

**Published:** 2021-01-14

**Authors:** Aranka Dol, Christina Bode, Hugo Velthuijsen, Tatjana van Strien, Lisette van Gemert-Pijnen

**Affiliations:** 1 Department of Psychology, Health and Technology, Faculty of Behavioural, Management and Social Sciences (BMS), University of Twente, De Zul 10, 7522 NJ Enschede, The Netherlands; 2 Institute for Communication, Media & IT, Hanze University, Groningen, The Netherlands; 3 Behavioural Science Institute, Radboud University, Nijmegen, The Netherlands

**Keywords:** Emotional eating, Obesity, Dialectical behavioural therapy, Personalized coaching

## Abstract

**Background:**

Around 13% of the world’s population suffers from obesity. More than 40% of people with obesity display emotional eating behaviour (eating in response to negative emotions or distress). It is an alternate to more effective coping strategies for negative emotions. Our study explored the opportunities for helping adults with emotional overeating using a virtual coach, aiming to identify preferences for tailored coaching strategies applicable in a personal virtual coach environment. Three different coaching strategies were tested: a validating, a focus-on-change, and a dialectical one – the latter being a synthesis of the first two strategies.

**Methods:**

A qualitative study used vignettes reflecting the two most relevant situations for people with emotional eating: 1. experiencing negative emotions, with ensuing food cravings; and 2. after losing control to emotional eating, with ensuing feelings of low self-esteem. Applied design: 2 situations × 3 coaching strategies. Participants: 71 adult women (*M*_*age*_ 44.4/years, range 19–70, SD = 12.86) with high scores on the DEBQ-emotional eating scale (*M*_*emo*_ 3.65, range 1.69–4.92, SD = .69) with mean BMI 30.1 (range 18–46, SD = 6.53). They were recruited via dieticians’ practices, were randomly assigned to the conditions and asked how they would face and react to the presented coaching strategies. Data were transcribed and a thematic analysis was conducted.

**Results:**

Qualitative results showed that participants valued both the validating coaching strategy and the focus-on-change strategy, but indicated that a combination of validation and focus-on-change provides both mental support and practical advice. Data showed that participants differed in their level of awareness of the role that emotions play in their overeating and the need for emotion-regulation skills.

**Conclusion:**

The design of the virtual coach should be based on dialectical coaching strategies as preferred by participants with emotional eating behaviour. It should be tailored to the different stages of awareness of their emotions and individual emotion-regulation skills.

## Plain English summary

Emotional eating is eating in response to negative emotions, and is problematic because it may lead to overweight, depression, and low self-image. People with emotional eating have difficulty regulating these emotions and are in need of mental healthcare but may feel too ashamed to seek help. Moreover, healthcare is not always available at the exact moment of the greatest coaching needs. Our goal in this research project is to develop a virtual coach application that is available 24/7.

This study examined what kind of automated coaching adults with emotional eating would prefer, and what they would consider as helpful to avoid emotional eating behaviour. Participants were asked questions to reveal their opinion about real-life situations, commented on by a virtual coach. They valued validation of their emotions, but believed a focus on behavioural change to be just as important. Participants displayed differences in emotional awareness of the association between overeating and regulation of emotions. These findings are of importance toward developing an adaptive personalized virtual coach that helps users in their struggle against emotional eating.

## Background

The fast growth of obesity is a threat to public health and around 13% of the entire world population suffers from obesity [1]. People with obesity often have physical, metabolic, and psychological comorbidities such as cardiovascular conditions, type-II diabetes, joint disorders, sleep apnea, and depression [2], and experience a lower quality of life [3, 4].

### Emotional eating

Between 40 and 60% of individuals with obesity have a high degree of emotional eating, defined here as a tendency to eat in response to distress or other negative emotions [5–7]. Emotional eating is associated with cravings for and intake of energy-dense food, and thus additional calories [8–11], binge eating [12], weight gain and, ultimately, obesity [13, 14]. Emotional eating is an atypical response to distress because the typical and adaptive response to negative mood such as feelings of depression is loss of appetite. Distress is normally associated with physiological responses that mimic the internal sensations associated with feeding-induced satiety, e.g. inhibition of gastric motility and release of sugar into the bloodstream [15]. It has been postulated that the atypical response of emotional eating develops early in life [16], as a possible outcome of parenting practices that inadequately met the child’s needs [17–19]. If parental responses to these needs are continuously inappropriate – be it neglectful, indiscriminately permissive, or over-controlling – the child may develop poor satiety awareness (deficient sentience of physiological symptoms associated with hunger and satiety), poor emotional awareness (resulting in difficulty identifying and describing emotions, also referred to as alexithymia) [20], and difficulty with regulating emotions [21, 22]. Poor satiety awareness and alexithymia were indeed shown to be associated with higher degrees of emotional eating [23–25]. A longitudinal study evidenced a significant serial mediation between parenting quality in infancy and emotional eating at 12 years *and* at 16 years through the two mediators, suppression of emotions and alexithymia [26].

Emotional eating is perceived as a strategy to regulate negative emotions [27], and there is some experimental evidence that it helps people with emotional eating reduce their negative emotions *during* food intake [28]. After an emotional eating episode, they can, however, be overwhelmed by feelings of shame and disgust over their behaviour [29]. These strong emotions can merge into a negative cycle and can cause relapse of the problem behaviour.

**Fig. 1:**
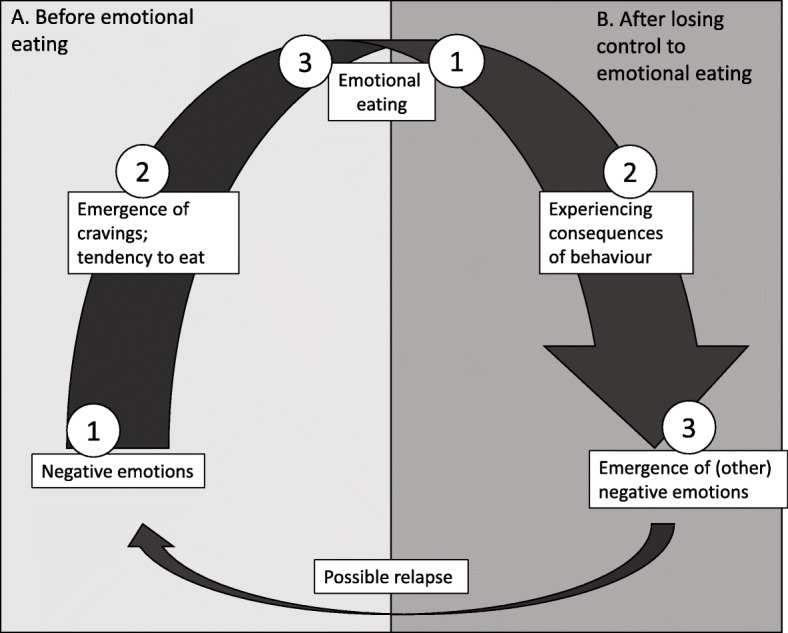
Model of emotional eating behaviour

According to our model of emotional eating behaviour (Fig. 1), there seem to be two tipping points at which interventions might be most effective: A. Before emotional eating, when experiencing 1) negative emotions and distress, and 2) cravings; and B. After giving in to emotional eating, when experiencing negative emotions (undergoing feelings of shame and disgust).

### Coaching strategies

There is increasing consensus that people with emotional overeating who are overweight are not helped by diets or cognitive behavioural therapy [30, 31], because these treatments focus on behavioural changes in food intake and physical activity instead of emotion-regulation abilities [32]. Instead, people with emotional eating benefit from learning to recognize, structure/restructure, and self-manage their emotions. Research shows that dialectical behaviour therapy (DBT) may be successful in treating emotional eating behaviour [33–39]. The coaching strategies within DBT are based on validation, focus-on-change, and dialectics – a fusion of the first two strategies [40].

People with emotional eating tend to be hard on themselves [41]. After each emotional eating episode they see confirmation of their own failure, with the ensuing shame and disgust [42]. Validation strategies suggest responding empathetically, by hearing the other person’s viewpoints and accepting them (and their emotions) without judging. Focus-on-change strategies present the receiver with a practical change-oriented focus on problem behaviour. Dialectics is a combination of acceptance of strong feelings and emotions on the one hand, and focus-on-change through adaptation of those feelings and emotions on the other [43].

### What are the coaching needs of people with emotional eating in relation to eHealth coaching?

Remoteness of healthcare providers and the need for self-management among people with emotional eating, as shown by previous research [44, 45], identifies the necessity for an online self-help tool: support that is in the hands of those with emotional eating, independently from place and time [46]. Our overall goal is to develop an adaptive virtual coach application that is available “24/7” – to assist in reducing emotional eating. This application is installed on the user’s smartphone. The user can ask the virtual coach for advice at any time and the virtual coach will respond with practical advice or exercises. To that end, in this qualitative study we examined the needs of users in terms of virtual coaching by presenting vignettes that mirror the most crucial situations and tipping points (see Fig. 1).

Preventive online coaching for people with emotional eating aims to provide support through a personalized virtual coach app. We apply human-centered design [47] as an approach to interactively develop a suitable and useful virtual coach. By involving users as stakeholders in all phases of the design process, the product will become a valuable and user-friendly intervention that fits users’ demands [48, 49].

The research questions for this article were:

1) Do adult participants with emotional eating behaviour identify themselves with the situations as presented in the vignettes [50]? (2) Which coaching strategy – validating, focus-on-change, or dialectical– matches the needs of adults with emotional eating “when experiencing cravings”, and to what extent is that strategy perceived as helpful by the participants? And (3) Which coaching strategy – validating, focus-on-change, or dialectical– matches the needs of adults with emotional eating “following the eating”, and to what extent is that strategy perceived as helpful by the participants?

## Methods

### Design

We presented participants with online vignettes containing lifelike scenarios of individuals with emotional eating [50], and invited them to preview the vignettes. Data were gathered by asking participants six questions about the scenarios in the vignettes. These questions offered both open-text and multiple-choice answer options (see Table 5 of Appendix). Participants’ personal eating style was assessed with the additionally filled in 33-item Dutch Eating Behaviour Questionnaire (DEBQ) [51]. This is a validated questionnaire with responses recorded on a 5-point Likert scale.

Using vignettes is an unobtrusive way for participants to give their opinion about a situation because it is about fictional persons and situations. Participants do not have to reveal their own personal emotions or eating behaviour. Vignettes have been found to be efficient in finding out people’s opinions about scenarios and individuals [52, 53]. The vignettes in this study described scenarios in the daily life of a person [54]: they contained a brief summing-up of the person’s demographic data such as name and age, followed by personal attributes like work and hobbies. The vignette also described the emotional state of the personas. The condition in the first vignette was about experiencing cravings and the urge to emotional eating. The second vignette addressed the condition in which the persona has just had an episode of emotional eating. In both situations the virtual coach responded with validating, focus-on-change, or dialectical coaching feedback on the given situation.

To validate the veracity of the vignettes, we assigned participants randomly to two conditions (“when experiencing cravings” and “after emotional eating”) with validation, focus-on-change, and dialectical coaching strategies originating from DBT [40, 43] (see Table 1).

**Table 1:** Distribution of vignettes (*N* = 144)

Condition	Persona	Attributes	Coaching strategy 1	Coaching strategy 2	Coaching strategy 3
1 – when experiencing cravings *N* = 67	Anita	Experiencing negative emotions or distress	Validating1	Focus-on-change1	Dialectical1
2 – after emotional eating *N* = 77	Lisanne	Low self-esteem and poor body image	Validating2	Focus-on-change2	Dialectical2

We asked the participants’ opinion on the described scenarios and the feedback given by the coach (for the provided feedback, see Table 6 of Appendix).

### Recruitment procedures

A pilot study was conducted among six participants (students) to validate the study protocol. To recruit participants, an information letter with an invitation to participate was sent out to all dieticians, who forwarded this invitation for voluntary participation to their clients. A hyperlink in the email directed applicants to an online survey. Initially, candidates were presented with information on the study, followed by an online letter of consent they had to agree with before proceeding to the first vignette. Candidates who did not give informed consent were excluded from the study. Due to the voluntary character of participation there were no consequences attached to the dietitian’s treatment. The dietitians didn’t know which of their clients participated in this vignette study.

Out of a total of 119 participants, 76 completed the entire study. It is not known why some participants did not complete the questionnaires. The participants, all female, had emotional eating, a BMI in adequate or high range, and were aged > 18 years.

### Participant features

The majority of participants were aged 40 to 60 years. Table 2 presents information on the participants’.

**Table 2:** Characteristics of participants (100% female)

Participants’ characteristics	Mean	Standard Deviation	Range
Age (years)	44	12.86	19–70
Weight (kg)	89	20.22	52–131
Height (cm)	170	7.00	150–185
BMI (weight/height^2^)	30.3	6.48	18–46
Eating style, emotional eating (DEBQ)	3.65 ^a^	0.69	1.69–4.92

^a^This score can be classified as “high” in comparison to the norm group of female age peers [6]

characteristics: 44% were obese with a BMI of 30–40, 35% were overweight with a BMI of 25–30, and 21%.

had an adequate BMI. Participants scored high on the DEBQ emotional eating scale, according to the norm tables for women aged 41–70 [6].

### Data analysis

All answers were sorted by the conditions “when experiencing cravings” and “after emotional eating”, and separately analyzed for the conditions. A thematic analysis according to Braun and Clarke [55] was conducted – a method combining top-down and bottom-up to find themes on a semantic level that can relate to the research questions by coding the raw data. The data was compared and collected in themes. Selective coding was conducted to gain deeper understanding of the themes and their interdependencies [56]. A reliable categorization was obtained by consensus-finding: the main coding was conducted by the first author. A colleague who is an independent scholar (N. de Jonge) was sent the coding table as well as the explanation of the codes and the corresponding examples, and coded a selection of 10% of the original data. We found a 100% agreement of codes with the first coder.

## Results

**Fig. 2:**
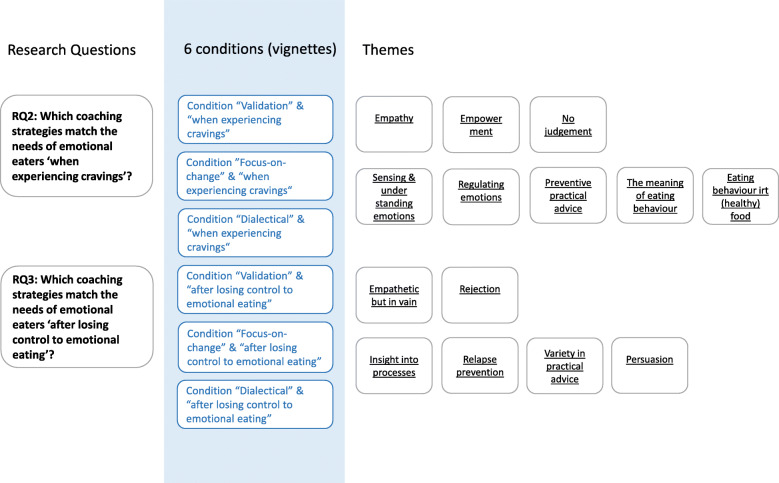
Navigation through the results of RQ2 and RQ3: an overview of the themes sorted into the six different conditions of the vignettes

This section presents the results by research question. The structure of the paragraphs for RQ2 and RQ3 is visualized in Fig. 2 below.

### RQ1: do participants with emotional eating behaviour identify themselves with the situations as presented in the vignettes?

The first question to the participants “*Do you identify yourself with the persona Lisanne or Anita?”* was meant to validate the given personas and scenarios (see Table 3).

**Table 3:** Results of the question ‘Do you identify yourself with … ’

Condition	emotional eating (lisval)^a^ *N* = 27	emotional eating (lisver) *N* = 24	emotional eating (lisdial) *N* = 32	Cravings (anval) *N* = 22	Cravings (anver) *N* = 24	Cravings (andial) *N* = 24
1 -Yes, totally	77.7%	58.3%	50%	68.2%	33.3%	41.7%
N	21	14	16	15	8	10
2 – Yes, more or less	22.2%	29.2%	34.4%	31.8%	54.2%	33.3%
N	6	7	11	7	13	8
3 -Yes, a little	0%	4.2%	12.5%	0%	8.3%	25%
N	0	1	4	0	2	6
4 – No, not at all	0%	8.3%	3.1%	0%	8.3%	0%
N	0	2	1	0	1	0

^a^***lis*** *Persona Lisanne,* ***an*** *Persona Anita;*

***val*** *= validating,* ***ver*** *focus-on-change,* ***dial*** *dialectical.*

Overall, nearly 90% of participants “totally” or “more or less” acknowledged all six conditions, i.e. “after emotional eating” (Lisanne persona) and “when experiencing cravings” (Anita persona) (55% totally; 34% more or less). For recognition, only 2.6% of respondents answered “a little” to “not at all”. The answer to RQ1 is that the numbers show a large portion of respondents recognizing themselves in the personas as presented in the vignettes.

### RQ2: which coaching strategy – validating, focus-on-change, or dialectical – matches the needs of people with emotional eating “when experiencing cravings”, and to what extent is that strategy perceived as helpful by the participants?

#### Condition “Validation & experiencing cravings”

Participants recognized two different forms of validation in this condition: validation of emotions and validation of behaviour.

##### Empathy

Participants experienced positive feelings and expressed that it feels good to get a compliment. They felt that the empathy offered by the virtual coach was warm and soothing. This also applied to participants that disapproved of their own eating behaviour.

*Very understanding. Anita immediately regains trust and she will not take the cookies either (anval-53).*

##### Empowerment

It reinforced their self-confidence. It accepted them as they were – the coaches’ support made them feel proud of themselves.

*Getting compliments about having made the right choice will increase your self-esteem and self-confidence. It will make it easier to stick to this feeling the next time (anval-53).*

##### Non-judgmental

Participants indicated that they didn’t see the point of validation, but still they appreciated it. It was a good feeling to be accepted as they really were, and their emotions were being taken seriously.

*It’s nice to talk to someone or to get a response when you’re about to eat. Nice that someone thinks along (andial-75).*

Validation of behaviour turned out to be a well-appreciated coaching strategy in a craving situation. It activated feelings of pride and delivered positive reinforcement because Anita (the persona in the vignette) was able to leave the cookies in the jar. It reinforced the decision taken. It was very supportive and confirmed and rewarded positive behaviour.

*Identifies good behaviour and states that she can feel proud by persevering (anval-53).*

#### Condition “Focus-on-change & experiencing cravings”

##### Sensing and understanding emotions

Participants noted that they would like to gain more knowledge about their own emotions and feelings. Not only did they want to learn about how to sense their own emotions, but they were also in need of more knowledge about how to deal with them. Instead of emotional eating it would be useful to reflect on the situation, pause, and discontinue the flow of behaviour in order to take a moment for reflection.

*I think that it is good to reflect on what you’re feeling (anver-64).*

##### Regulating emotions

Most participants were well aware of their emotions. They mentioned the desire to influence their emotions and consequently gain more control over their eating behaviour. They wanted to break behavioural patterns and recurring thoughts, and were already familiar with their own personal emotional eating behaviour. Participants were totally aware of what was going on, but wanted to learn how to cope with their emotional distress and deal with it, instead of ‘giving in’ to emotional eating.

*Providing advice about the emotions; explaining that it isn’t necessary to eat just because others are doing it; and ways to deal with stress, other than the cookie jar (anver-67).*

##### Preventive practical advice

Most participants indicated preferring advice, with practical tips on how to prevent or stop their undesirable eating behaviour. They pointed out that what they need is a reach-out – someone who holds their hand and suggests practical steps. Participants wanted solutions on how to anticipate and recognize signals that the cravings they are experiencing may be harbingers of emotional eating. They expressed a need for practical advice in the form of action plans or roadmaps; one suggestion was making a relapse prevention plan by coming up with substitute activities. In this way temptation is resisted and the impulse to eat suppressed, developing alternative habits and at the same time avoiding difficult situations.

##### The meaning of eating behaviour

Participants wanted to understand out what lies behind the emotional eating behaviour, and find out what food stands for. They wanted to gain better insight into the behaviour and the conditions that lead to eating.

*Try to figure out what is really going on, what does “food” stand for? (andial-78).*

##### Eating behaviour in relation to healthy food

The need for practical tips often focused on food and health. Participants never mentioned diets or lifestyle programs, yet expressed a need for education on planning their meals and how to provide for satisfying and wholesome meals. They wanted to avoid the “bad” foods and learn to find alternatives for sweetmeats and snacks.

*Would like to know what food a body needs to function properly and what, for example, too much sugar can do to your body (anval-56).*

#### Condition “Dialectical & experiencing cravings”

As stated earlier, participants felt positive about both the validation and the focus-on-change coaching strategies. However, the answers revealed that most participants identified *dialectical* as the preferred coaching strategy. They liked the idea of an understanding, positive approach and empathy, and the dialectical strategy is also focused on solutions.

*This one is supportive, but … I would like more concrete help or instructions (anval-53).*

*Compliments and advice on how to keep your self-esteem at moments like this (anval-56).*

*Sympathetic, but also tips on how she can do even better. Like giving her “homework” (andial-77).*

### RQ3: which coaching strategy – validating, focus-on-change, or dialectical – matches the needs of people with emotional eating “after losing control to emotional eating”, and to what extent is that strategy perceived as helpful by the participants?

#### Condition “Validation & after losing control to emotional eating”

##### Empathetic, but in vain

Participants reported having experienced empathy and acknowledgment of their emotions. It was perceived as positive and not disapproving, but it did not provide participants with practical tips on how to act differently next time.

*It is empathetic and non-judgmental, but is not offering anything to prevent a binge next time (lisval-18).*

##### Comfort in food

The reaction of the coach – confirming that eating is soothing – was unanimously declined: participants expressed that food can never be comforting. They did not tolerate condonement of emotional eating behaviour, and stated that finding comfort in food or in eating is impossible.

*The coach justifies eating behaviour. That isn’t very helpful (lisval-18).*

*You will find no comfort in food (lisval-18ng).*

#### Condition “Focus-on-change & after losing control to emotional eating”

##### Insight into processes

A significant number of participants indicated being in need of deeper insight into the whole emotional process, in order to break through patterns and habits. They said it would be useful to have the virtual coach question them about situations and occurrences that preceded the emotional eating, relating these events to feelings and emotions that were predominant. Participants wanted the virtual coach to ask them questions, so they could learn to analyze and influence or regulate their emotion or behaviour, as well as apply that knowledge to reducing the behaviour.

*Try to find out for yourself what happened today, and try to recall how you felt at that particular moment (lisdial-44).*

##### Relapse prevention

According to the participants, the virtual coach should confront them with questions such as: “*What are your thoughts? Are they true? How are you so sure?”*. The coach should ask more in order to gain better insight into an individual’s eating behaviour, helping them reconstruct what just happened and make a relapse prevention plan.

*How can I prevent this next time? Can I do something that doesn’t make me feel so bad that I will give in to bingeing, or to make me react differently when I feel bad? Perhaps – if I cannot prevent a binge – advice on alternative, less harmful food? Tips how to get out of the binge. (lisval-21).*

*What she can do the next time she feels like she’s caving to bingeing, for example experiencing her emotions or seeking a distraction (lisval-21).*

##### Variety in practical advice

Participants were in need of guidance and advice. They were on the lookout for alternative activities, for distractions that could lead away from the cravings or from the eating that just took place, or even for an escape from a binge while being in the middle of one. Others suggested the virtual coach could provide encouragement to take action.

*How can I prevent this next time, and where can I go for help? (lisver-33).*

##### Straightforwardness

Some participants reported a preference for a more confrontational coaching style. They wanted the coach to be friendly and empathetic, but at the same time “put it bluntly” and get straight to the point that it’s all about the eating behaviour.

*Straightforwardly with direct advice, so that you have tools ready for you to move on (lisval-20).*

#### Condition “Dialectical & after losing control to emotional eating”

Participants liked the concept of an understanding, positive approach and empathy (validation), but were also very clear in their need for additional practical advice. Empathy is warm and soothing, but is not going to “get you anywhere”. Participants observed that the coach offers a message consisting of both positive feedback and a reach-out toward change. The majority of participants preferred this combination of validation with a focus-on-change approach as being the best coaching strategy.

*The coach confirms to Lisanne what she feels, showing a way out of the emotion. It might be useful to give a hint about what she can do next time to prevent a binge (lisver-30).*

*The response is supportive and non-judgmental. The reaction of the coach also points to taking immediate action. I think that’s a good thing, the idea that you could immediately move on in a positive manner. That should not be tomorrow, or the day after tomorrow, but now (lisdial-42).*

Selective coding was conducted to get a deeper understanding of the themes and their.

interdependencies. The data revealed that people with emotional eating are at different levels of their emotions and emotion-regulation, and that they might be divided into three groups:

1. People who just want a practical solution for the problem; they are unaware of the association between their emotions and their eating behaviour.
2. People who are aware of their emotions and feel an unclear association between their emotions and their eating behaviour, but do not express it in terms of emotion-regulation.
3. People who are aware of their emotions, and express the need to learn how to regulate them.

Common elements in all these groups were the cultivation of more positive emotions to reduce negative emotions, and learning to gain insight into one’s own feelings.

## Discussion

**Fig. 3:**
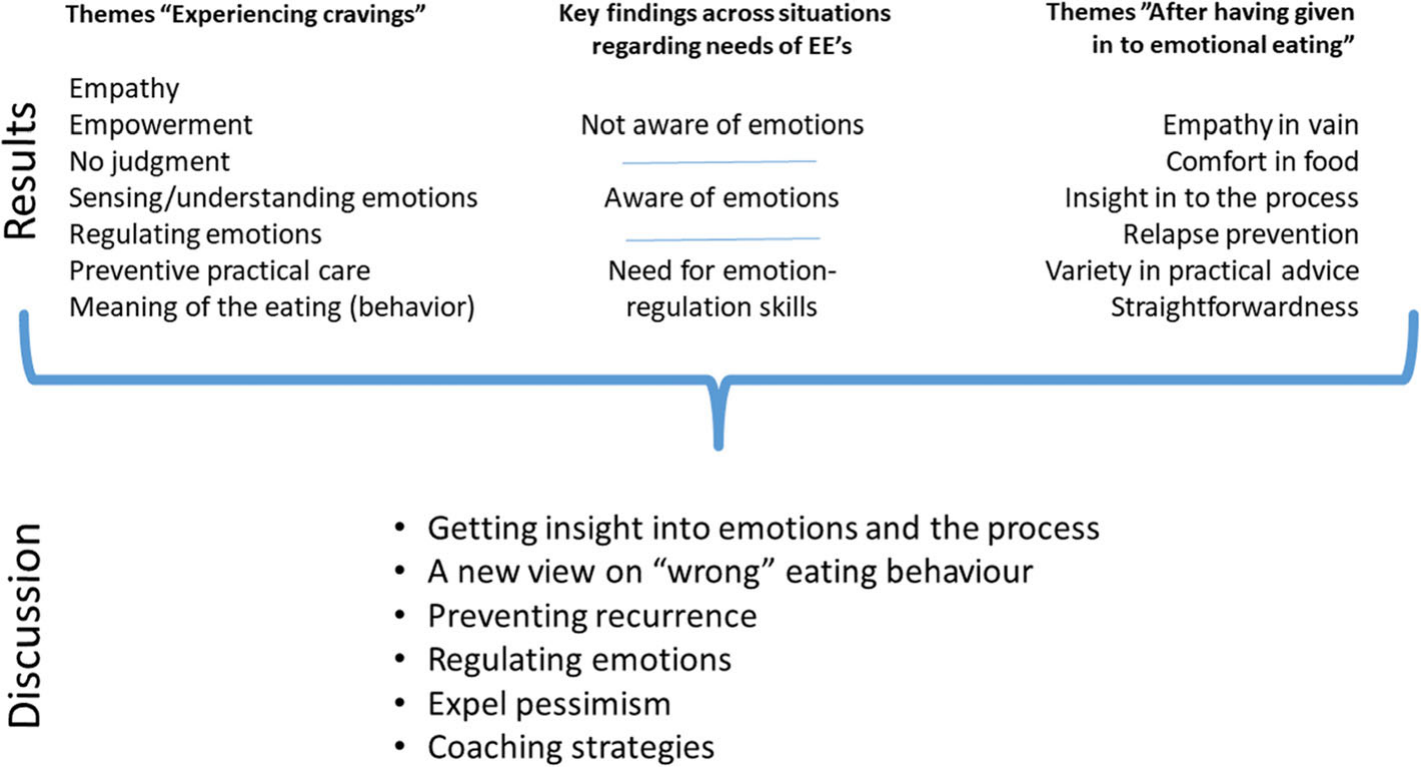
Overview of found themes per condition (“When experiencing cravings” and “After losing control to emotional eating”) translated to topics in the Discussion

The objective of this study was to get a clearer view of what kind of coaching strategy is preferred by people with emotional eating” when experiencing cravings” or” after losing control to emotional eating”. The main findings were that participants identified themselves with the personas and that they valued both the validating coaching strategy and the focus-on-change strategy. At the same time, they indicated that a combination of validation and focus-on-change – the dialectical coaching strategy – was appreciated the most. Below we present a summary of results related to literature and conditions for tailoring the virtual coach to the needs of individuals with emotional eating tendencies (Fig. 3).

### Gaining insight into emotions and the process of emotional eating

Participants in our study indicated having difficulties identifying and describing their feelings and communicating these feelings to others. This is in line with earlier research establishing an association between emotional eating behaviour and alexithymia [57–62]. Participants also pointed out their need for deeper insight into the entire process of emotional eating. A possible way for participants to have a better comprehension of the possible triggers and causes of their individual bouts of emotional eating could be by inviting them to conduct a cause-effect analysis of the chain of events that ultimately lead to emotional eating behaviour. Such analysis could help people with emotional eating identify and describe their feelings, as well as provide essential information for understanding the events and the resulting emotions that led up to the problem behaviour [40]. Chain analysis is a powerful tool in DBT [63]. By conducting repeated chain analyses a person can identify the pattern linking different components of a behaviour. These analyses were an integral part of several studies on people with binge-eating disorder (BED) or eating disorders in general [33, 64–70]. The publications indicated that the deployment of dialectical behaviour therapy in these target groups can undoubtedly be considered successful, but unfortunately the effect of specific deployment of the chain analysis was unknown. The virtual coach could provide tools to help users analyse the entire chain of cause-and-effect. Such a self-management tool can be used as a stand-alone or as a preparatory blended care element in DBT chain analysis [40, 50].

### A new view on “wrong eating behaviour”

The majority of participants disapproved of validating the eating behaviour “after emotional eating”. The fact that the virtual coach showed understanding for the person finding comfort in food at the end of a disappointing day was considered unacceptable. The person’s behaviour was deemed “wrong” by the participants. Feelings of guilt may have predominated [71, 72] – participants apparently considered the emotional eating behaviour to be a greater transgression than allowing themselves the ephemeral comfort of food.

Our results revealed that participants did not consider food as something comforting. Most people are brought up with the notion of comfort food – consumed in periods of distress, evoking positive emotions and associated with “significant social relationships” [73] or “a specific food consumed under a specific situation to obtain psychological comfort” [74]. The same holds for people with a high degree of emotional eating, who according to Van Strien derive comfort from palatable food [28]. Consumption of highly palatable food induced by negative emotions stimulates the reward center [59], albeit only on a temporary basis. Research among people with binge eating disorder shows that any potential positive affect decreases as soon as the binge episode ends [75].

And this is where the paths appear to separate: where comfort food leaves most people with feelings of contentment, people with emotional eating will feel overwhelmed by feelings of shame and regret, and consequently possible relapse [76]. Feelings of regret are probably triggered by the amount of food that was consumed. Such individuals consume more energy-dense foods in response to negative emotions than people without emotional eating [10]. Plus, eating comfort food may not fit within their regime of calorie restriction, so any violation of this regime will cause them to be dissatisfied with their lack of discipline or steadfastness.

An additional problem is that people with emotional eating eat out of sight of their social network. Obviously, emotional eating behaviour does not meet the norms of social eating behaviour [77, 78], and due to feelings of shame people with emotional eating are not able to share their thoughts in a safe environment such as friends or family. A possible role for the virtual coach is to provide help restoring their relationship with food where eating is pleasant, and preferably with the companionship of friends and family.

### Preventing recurrence

The majority of participants identified a need for practical advice on how to prevent the eating behaviour. They wanted solutions to how to anticipate and recognize signals when experiencing cravings. Participants pointed out that they have a need for distraction – what to do instead of eating. Research shows that different forms of distraction lead to changes in the desire to eat, but some activities such as watching television were associated with mindless eating behaviour and increased food intake [79]. During times of social interaction there was no urge to eat. According to Crockett [80], both proneness to boredom and emotion-regulation strongly correlated with the emotional eating variables. Participants’ suggestions included an action plan that can propose alternative activities, as well as a relapse prevention plan that provides the user with distracting thoughts and statements. In this way temptation is resisted and the impulse to eat suppressed. It helps the user avoid difficult situations and develop alternative habits.

There is a large body of literature regarding relapse prevention plans in relation to addiction, as presented by Marlatt & Donovan [81]. Such a plan often serves as an extension to an existing therapy, like maintenance strategies as post-treatment for obesity [82]. The participants in this study may benefit from setting up their own prevention plan in the virtual coach. Bauer & Moessner [83] showed that it is certainly possible to offer technology-enhanced relapse prevention, even though its effectiveness has not yet been widely demonstrated. Ideally, the prevention plan should be in line with the chain analysis described above, in which users learn to recognize difficult situations and identify their own pitfalls. By using the coach they themselves would learn to recognize their own relapses.

### Regulating emotions

Unlike the participants described above under *“Gaining insight into emotions and the process”* (those who suffer from the inability to express their emotions), a vast majority of participants were well aware of their inability to deal adequately with their emotions and assuage them. Knowing that eating is their coping strategy to handle negative emotions, participants expressed a wish to learn how to deal with emotional distress. They were in need for instructions and tools to regulate agitation, stress and anger. When negative affect is experienced and people regulate it maladaptively, this inadequate emotion-regulation strategy can be responsible for increased eating [84, 85]. Literature shows that there is a link between being able to regulate one’s emotions and level of emotional awareness [86]. Emotional awareness seems to be a requisite for a person’s emotion-regulation skills [87]. A potential task for the virtual coach would be to fulfil these requirements by providing information and exercises on regulation of emotions.

### Expelling pessimism

A majority of participants saw themselves as unable to break through fixed patterns without help. They expressed the need for exercises that would help improve their basic attitude and bring about behavioural change. The results of this study are in accordance with previous findings. People with emotional eating seem to be pessimistic and have a negativity bias [88]. Not only is their thinking distorted, but by overestimating the chances of bad things happening to them [89] they also tend to disqualify the positive and instead have negative emotions and thoughts [90]. Szczygieł’s study shows that the potentially damaging impact of negative emotions on the processing of emotional information can be prevented by high emotional awareness or by implementing reappraisal as an emotion-regulation strategy [91].

Further, poor self-control is related to emotional eating induced by negative emotions [92, 93]. Together with the negativity bias, there is a reasonable likelihood that people with emotional eating might relapse, as illustrated in Fig. 1. The virtual coach could provide users exercises from cognitive restructuring to learn to accumulate positive experiences in life [94] or offer positive reappraisal [95].

### Coaching strategies

Participants displayed poor self-image. It has been described in the literature that people with emotional eating are hard on themselves. They have a negative body image, [96–99] articulated by body hate – negative expressions about their own body and body parts. Individuals with alexithymia score higher in body dissatisfaction and have lower self-esteem [100]. After an emotional eating episode, people may be overwhelmed with feelings of disgust and low self-esteem [76].

Even though participants had a negative attitude toward themselves, a vast majority felt positive about the validating coaching strategy. The fact that their true feelings and emotions were expressed by the coach could be considered as meeting expectations that lie in their unspoken needs. The coach describes emotions and reveals what is considered as “terra incognita” for the participants. The majority of participants disapproved of validating the eating behaviour “after emotional eating”, which concerns the comforting aspect of eating. At the same time, this study has made it clear that participants showed gratitude for not being judged on their eating behaviour. This may indicate an unspoken need for a counterargument, something they aren’t able to give themselves. Validation of their behaviour yielded an opposite point of view, and can teach them to be more lenient in judging their own behaviour. According to Swales [101], “Acceptance implies an acknowledgement of what is rather than approval or agreement” (p.166), and “Validation helps clients tolerate the extreme difficulty of change”. The virtual coach can meet the need to enhance self-compassion by providing validating coaching strategies for self-image.

Participants appreciated the straightforwardness and practical approach of the dialectic coaching strategy. It not only validated their emotions and behaviour – the approach made them feel accepted for who they are. At the same time they received solution-oriented advice on the best thing they could do in that specific situation.

Although the effectiveness of DBT in both BED and emotional eating behaviour has been extensively published [33, 64–70, 102], little is known about the effect of the specific coaching strategies that are a part of this therapy [43, 102]. Further research is needed on which type of validating or dialectical coaching fits best with which situation.

When combining the needs articulated in our study with the two most important situations for people with emotional eating (“when experiencing cravings” and “after losing control to emotional eating”), we propose the following scheme to plan and design exercises tailored to specific situations and personal coaching needs (see Table 4).

**Table 4:** Subgroups of future users sorted by type of awareness, combined with potential interventions of the virtual coach

Subgroups of users	Possible intervention “when experiencing cravings”	Possible intervention “after emotional eating”
1. Is unaware of emotions	*Gaining insight into emotions and the process: how to stop automatic behaviour; monitoring emotions and analyzing the cause-and-effect chain with the emotion analyzer* [50]	*A new view on “wrong eating behaviour”: restoring the relationship with food of people with emotional eating*
2. Is aware of presence of emotions but unaware of need for emotion-regulation	*Preventing recurrence: relaxation (how to release the emotion; distraction); replacing eating with other activities*	*Coaching strategies: adjusting one’s self-image; validating emotions*
3. Is aware of emotions and of need for emotion-regulation	*Regulating emotions: learning to endure distress; nudging to think again*	*Expelling pessimism: cognitive restructuring; fighting pessimism and negativity bias.*

### Strengths and limitations of the current study

The vignette methodology worked well to present participants with real-life personas and situations they could identify with. We tested this by asking participants whether they could relate to the presented real-life personas. The recognisability of the content in their own daily life created a safe and valid environment to reflect on the stories. Participants were representative of a clinical sample of people with elevated levels of emotional eating, therefore the results have external validity and the interpretations could be implemented more directly into virtual coaching practices of people with emotional eating. A limitation of the study was that the participants were all women, which implies that the results can only be applied and generalized to female clients of virtual coaching.

### Future research

This study provided us with information on how coaching should be designed for female adults with emotional eating. Future studies would need to clarify how to design the coach’s support facilities in order to serve different types of users depending on their level of knowledge and awareness of emotions and emotion-regulation skills.

## Conclusion

Qualitative results showed that people with emotional eating clearly prefer dialectical coaching in both the “when experiencing cravings” and “after emotional eating” conditions. Our study revealed that the participants were at different stages of knowledge and awareness about the role of emotions in their eating behaviour and emotion-regulation abilities. Vignettes showed to be effective in finding out opinions of people with emotional eating about their coaching preferences under different conditions. The given results allow us to make the first attempt to define what particular kind of virtual coaching people with emotional eating need in specific circumstances, but additional research is needed to collect monitoring information [103] in order to personalize the coaching as precisely as possible and keep users motivated in the long term.

## Data Availability

The qualitative datasets (i.e. transcripts, coding, and themes) used and/or analyzed during the current study are available from the corresponding author upon reasonable request.

## Appendix

**Table 5:** Questions about the vignettes

Questions to the participants	
1. Do you identify yourself with the persona Lisanne/Anita? Answers (2 mc^a^): Yes, I can totally relate to L/A. Yes, I can relate more or less […]. No, I cannot relate very well […]. No, I cannot relate at all […].	
2. What is your opinion of the coach’s reaction to L’s/A’s problem? Answers (2 mc + free text): I think that is a good response, because … I don’t think that is a good response, because …	
3. What would you think if you would got an answer like this yourself? Answers (5 mc + free text): I find it helpful, that’s what works for me. I find it friendly, supportive, I feel understood. I find it too confrontational. I find it useless. Other (explain):	
4. What kind of advice would you give to L/A if you were the coach? Answers (4 mc + free text): Be empathetic, offer words of comfort. Just put it bluntly – no beating around the bush. Advice on what to do. Other (explain):	
5. What would you ask the coach if you were in a similar situation? Answers: free text	
6. What kind of advice would you like to get from a coach? Answers (4 mc + free text): Be empathetic, get words of comfort. Just put it bluntly – no beating around the bush. Advice on what to do. Other (explain):	

^a^*mc* Multiple-choice

**Table 6:** Provided feedback per condition, per coaching strategy

Condition	Coaching strategy	Provided feedback
1	Validation	*‘Well done Anita, that you left the cookies in the tin. I do know how difficult that is. You have every reason to be proud of yourself.’*
2	Validation	*‘Hi Lisanne, so sorry you had a binge. I understand that you had a craving for comfort food after a day like this. It is quite understandable you feel awful now.’*
1	Focus-on-change	*‘Hi Anita, try to hang on to the feeling you are experiencing right now, and try to recall it next time you’re about to give in to eating.’*
2	Focus-on-change	*‘Hi Lisanne, so good you contacted me! Don’t be angry with yourself, it takes time to make a change. Grant yourself that time. What could you do to get rid of that bad feeling and move on from there?’*
1	Dialectical	*‘Hi Anita, so good you didn’t touch the cookies. I know how hard that is. You have every reason to be proud of yourself. Try to stick to the feeling you are experiencing right now, and try to remember that right now you are about to cave to snacking.’*
2	Dialectical	*‘Hi Lisanne, so sorry you had a binge. After a day like this, you obviously feel a need to eat and feel comforted. Don’t be angry with yourself. The challenge now is to take good care of yourself. What could you do to get rid of that bad feeling and move on from there?’*
